# Proteomics and Lipidomics to unveil the contribution of PCSK9 beyond cholesterol lowering: a narrative review

**DOI:** 10.3389/fcvm.2023.1191303

**Published:** 2023-06-12

**Authors:** Erica Gianazza, Chiara Macchi, Cristina Banfi, Massimiliano Ruscica

**Affiliations:** ^1^ Unit of Functional Proteomics, Metabolomics and Network Analysis, Centro Cardiologico Monzino IRCCS, Milan, Italy; ^2^ Department of Pharmacological and Biomolecular Sciences “Rodolfo Paoletti”, Università degli Studi di Milano, Milan, Italy; ^3^ Department of Cardio-Thoracic-Vascular Diseases, Foundation IRCCS Ca’ Granda Ospedale Maggiore Policlinico, Milan, Italy

**Keywords:** proprotein convertase subtilisin/kexin type 9, atherosclerotic cardiovascular disease, proteomics, lipidomics, extracellular vesicles

## Abstract

Proprotein convertase subtilisin/kexin type 9 (PCSK9), one of the key regulators of the low-density lipoprotein receptor (LDLR), can play a direct role in atheroma development. Although advances in the understandings of genetic *PCSK9* polymorphisms have enabled to reveal the role of *PCSK9* in the complex pathophysiology of cardiovascular diseases (CVDs), increasing lines of evidence support non-cholesterol-related processes mediated by PCSK9. Owing to major improvements in mass spectrometry-based technologies, multimarker proteomic and lipidomic panels hold the promise to identify novel lipids and proteins potentially related to PCSK9. Within this context, this narrative review aims to provide an overview of the most significant proteomics and lipidomics studies related to PCSK9 effects beyond cholesterol lowering. These approaches have enabled to unveil non-common targets of PCSK9, potentially leading to the development of novel statistical models for CVD risk prediction. Finally, in the era of precision medicine, we have reported the impact of PCSK9 on extracellular vesicles (EVs) composition, an effect that could contribute to an increased prothrombotic status in CVD patients. The possibility to modulate EVs release and cargo could help counteract the development and progression of the atherosclerotic process.

## 1. Biology and functional roles of PCSK9

Since its discovery in 2003, as the third gene critical for low-density lipoprotein cholesterol (LDL-C) regulation (1), increasing lines of evidence supported non-cholesterol-related processes mediated by PCSK9 (proprotein convertase subtilisin/kexin type 9). Although mainly derived from the liver, PCSK9 is expressed in many tissues (e.g., intestine, pancreas and central nervous system) and cells types (e.g., macrophages) (2–4).

PCSK9 is the ninth member of a family of endoprotease enzymes. Seven of the nine are core members that are structurally and biochemically similar to each other and to the bacterial (subtilisin) and yeast (kexin) proteins from which they were derived. Proprotein convertases (PC) are synthesized as zymogens (inactive precursors) that are chaperoned through the cell by their prodomains. These contribute to protein folding and activation, favouring the egress of the core proprotein convertases from the endoplasmic reticulum (5). Differently from the first seven members of the PC (PC1, PC2, furin, PC4, PC5, paired basic amino acid cleaving enzyme 4 (PACE4) and PC7) (6) PCSK9 cleaves itself at its internal VFAQ152↓ sequence, and then no longer functions as a protease is allowed. PCSK9 acts as a binding protein to specific cell surface receptors and in particular fosters the endosomal and lysosomal degradation of LDL receptor (LDLR) (7). The three-dimensional structure of PCSK9 revealed the existence of three distinct structural domains, the prodomain (aa 31–152), the catalytic subunit (aa 152–421), and the C-terminal Cys/His-rich domain (aa 453–692), each playing critical roles in the regulation of PCSK9 and its intracellular trafficking (8, 9). Degradation of the LDLR by PCSK9 depends on the internalization of the PCSK9-LDLR complex into clathrin-coated acidic endosomes. Through its catalytic subunit, secreted/plasma PCSK9 binds the epidermal growth factor precursor homology domain A (EGF-A) of the LDLR fostering its degradation in endosomes/lysosomes, rather than being recycled (10). In particular, Gly^293^, Asp^299^, Arg^329^, and Glu^332^ in EGF-A of the LDLR are pillar to PCSK9 binding at the cell surface (11).

### 1.1. Preclinical evidence of atherothrombosis

The expression of D374Y gain-of-function variant of human PCSK9 in mice has led to accelerated atherosclerosis, a process beyond the reduced activity of LDLR (12). Conversely, the lack of PCSK9 was linked to the reduction of neointima formation in atherosclerotic plaques in full knockout mice (13). Similar con-clusions were raised by Sun et al. showing that hepatic reduction of PCSK9 affects atherogenesis by modulating the levels and properties of apolipoprotein B-containing lipoproteins, an effect independent of PCSK9-driven modulation of LDLR (14).

**Figure 1:**
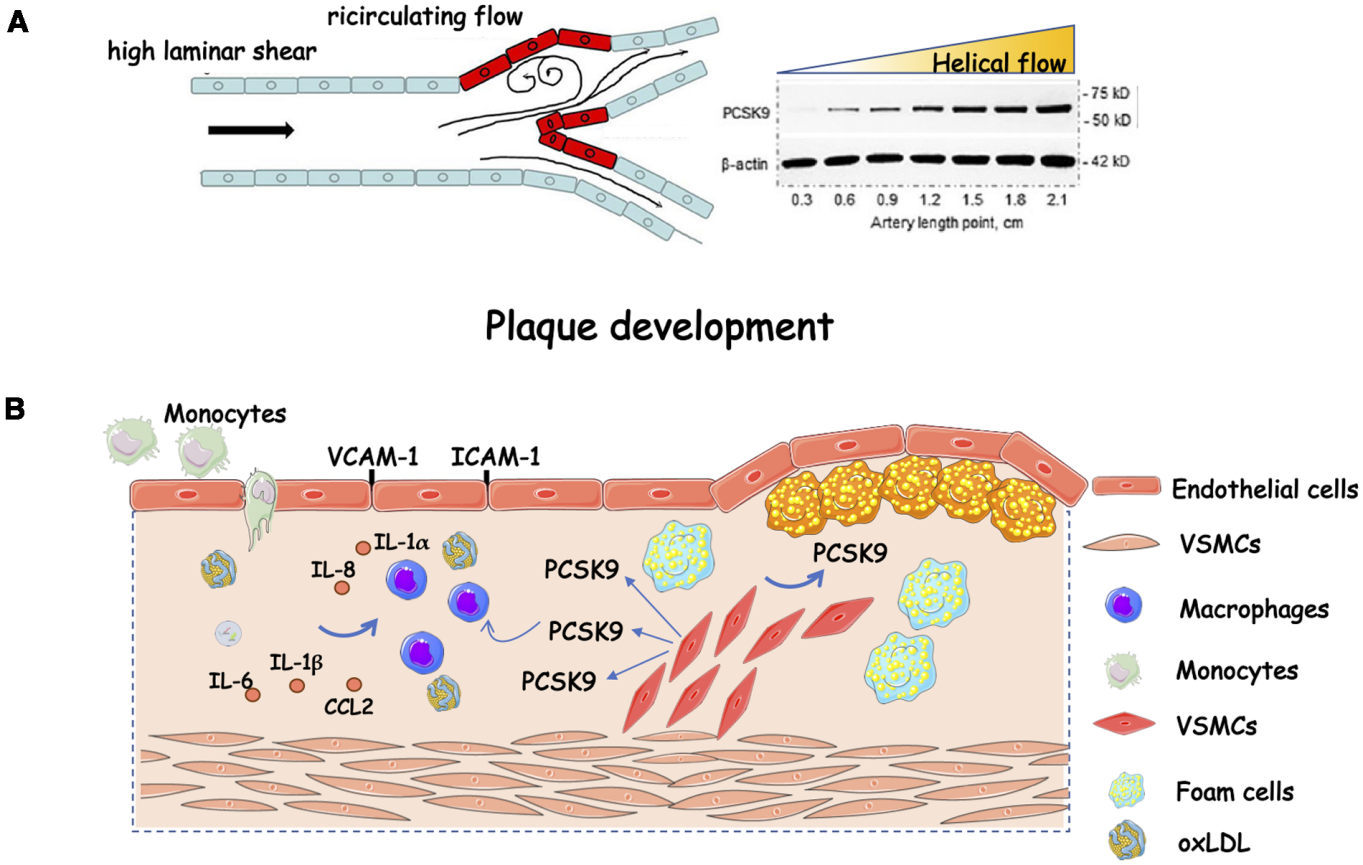
Role of PCSK9 in plaque progression. (**A**) Helical flow inhibits whereas low flow induces protein PCSK9 expression; (**B**) Schematic representation of the effect of PCSK9 on plaque progression fuelling. Compelling evidence implicates PCSK9 in plaque development. It is expressed in endothelial cells, vascular smooth muscle cells, and macrophages. In these latter, there is a stepwise increase in PCSK9 gene expression while transitioning from monocytes, to differentiating monocytes, to fully differentiated macrophages. Exposure to human recombinant PCSK9 up-regulates the mRNA expression of proinflammatory cytokines and chemokines (e.g., IL-1β, IL-6, TNF-α, CXCL2, and monocyte chemoattractant protein-1). CCL2, C-C motif chemokine ligand 2; ICAM-1, intercellular adhesion molecule 1; IL, interleukin; oxLDL, oxidized low-density lipoprotein; VCAM-1, vascular cell adhesion protein 1; VSMCs, vascular smooth muscle cells. The figure has been partially modified by permission of Oxford press (30).

Mainly produced by the liver (15), circulating PCSK9 may enter the intimal space (16), where it may stimulate macrophages to produce proinflammatory cytokines and vascular smooth muscle cells (VSMCs) to migrate. In particular, PCSK9 seemed to promote M1 macrophage polarization at least *in vitro* (17). Migration of VSMCs from the media into the intima can contribute to the accumulation of VSMCs in the growing atherosclerotic plaque. These cells can proliferate over the years and elaborate extracellular matrix macromolecules that comprise much of the bulk of an established atherosclerotic plaque. Atherosclerotic macrophages and vascular smooth muscle cells also secrete PCSK9 within the plaque, contributing to increased inflammation and fatty deposition (18, 19). Chronic inflammation is a hallmark of atherosclerotic cardiovascular disease contributing to the initiation, progression and rupture of an atherosclerotic plaque (20). Within this context, the overexpression of human *PCSK9* in mice altered plaque morphology and increased the infiltration of inflammatory bone marrow-derived Ly6Chigh monocytes into the lesion, leading to their differentiation into macrophages (21). Pro-inflammatory stimuli such as tumour-necrosis factor-α and lipopolysaccharides (LPS) enhance the expression of PCSK9 in vascular endothelial cells and VSMCs (22, 23). In line with this evidence, the expression of PCSK9 was increased in mouse macrophages after stimulation with LPS, an effect mediated by the involvement of NLRP3 (NOD-Like Receptor Protein 3) inflammasome (24). Conversely, PCSK9 has been considered a trigger for the expression of pro-inflammatory cytokines, e.g., it raises the inflammatory milieu in macrophages (25). The overexpression of *PCSK9* in *Ldlr^−/−^* mice induced pro-inflammatory macrophage activation, namely, immune responses, proliferation, and migration, all via LDLR-independent mechanisms (26). Accordingly, a lower number of macrophages and vascular inflammatory regulators was found in atherosclerotic lesions of mice injected with short hairpin RNA targeting *PCSK9* (27). However, it is worth mentioning that haemodynamic shear stress, a major determinant of atherogenesis, affects the expression of PCSK9. A greater expression was found in aortic arch branch points and aorta-iliac bifurcation regions where the shear stress is low, than in the thoracic aorta and iliac arteries where shear stress is higher (23) (Figure 1). Considering that the role of platelets in atherothrombosis cannot be overlooked, *Pcsk9^−/−^* mice were protected from occlusion of the carotid artery induced by the topical application of FeCl_3_. Roughly 70% of *Pcsk9^−/−^* mice formed unstable nonocclusive thrombi whereas 57% of *Pcsk9^+/+^* littermates developed a total occlusion with stable thrombi (28). In line with these findings, PCSK9 enhanced platelet activation in a CD36 receptor-manner (29).

### 1.2. Clinical evidence of atherothrombosis

Carriers of loss-of-function (LOF) variants Y142X and C679X leading to ∼40% reductions in LDL-C and those carrying R46L leading to 15% reduction in LDL-C showed, respectively, astonishing drops of 88% and 47% in the incidence of coronary heart disease during a 15-year follow-up period (31). E144K and C378W mutations are two new LOF mutations that impair the self-cleavage and endoplasmic reticulum exit of PCSK9, respectively, thus preventing PCSK9 secretion and PCSK9-induced degradation of the LDLR (32).

A genome-wide association study and a following Mendelian Randomization analysis, conducted on a cohort of 3,290 individuals of the LIFE-Heart study receiving coronary angiography for suspected coronary artery disease (CAD), with a mean age of 61.7 years, supported the hypothesis of a causal effect of PCSK9 on coronary artery disease status and severity, carotid plaques, and intima-media thickness (33). A different conclusion was reached in Caucasian carriers of the T allele at R46l (34). In this PROSPER (Prospective Study of Pravastatin in the Elderly at Risk) study, 5,783 elderly participants were enrolled with an age between 70 and 82 years, of whom 43% had a history of vascular disease at baseline and randomized to pravastatin or placebo with follow-up. In these individuals, the minor allele at the R46l locus in *PCSK9* did not significantly reduce coronary heart disease or vascular disease risk. The ATHEROREMO-IVUS (The European Collaborative Project on Inflammation and Vascular Wall Remodeling in Atherosclerosis—Intravascular Ultrasound) study, performed on 581 patients (mean age 61.5 years) who underwent coronary angiography for acute coronary syndrome (ACS) or stable angina, showed that PCSK9 levels were linearly associated with higher necrotic core fraction in coronary atheromas (35). In line with these findings, a positive association was found between PCSK9 levels and a 10-year progression of carotid atherosclerosis beyond LDL-C (36). Conversely, neutral conclusions were reached when the relationship between serum PCSK9 concentrations and measures of vascular health, and subclinical atherosclerosis were assessed in a cohort consisting of 1,527 middle-aged men (mean age 49.4 years), who were in good health and free of vascular disease, enrolled in the Firefighters and Their Endothelium (FATE) study (37).

PCSK9 was also associated with arterial stiffness (38), an index linked to the presence of carotid plaques (39). This evidence was supported in two cohorts of Italian ancestry in which short-term therapy with monoclonal antibodies improved endothelial function (40) and arterial stiffness (41). Pertaining to platelet activation, the PCSK9-REACT study (Association of PCSK9 Serum levels and Platelet Reactivity in Patients with Acute Coronary Syndrome Treated with Prasugrel or Ticagrelor), performed on consecutive ACS patients undergoing percutaneous coronary intervention with a mean age of 57 years, found a positive association between circulating PCSK9 and platelet reactivity in patients with ACS undergoing percutaneous coronary intervention (42).

PCSK9 is also recognized as an atherothrombotic risk factor because is involved in inflammatory cytokines production, endothelial dysfunction, atherosclerotic plaque formation and rupture, as well as in the pathogenesis of atherothrombosis (43). PCSK9 promotes platelet activation and aggregation, leukocyte recruitment and clot formation (44) and a positive association between circulating PCSK9 levels and platelet markers exists (28, 42, 45). Besides that, the presence of PCSK9 on platelet was found in thrombi aspirated from the coronary arteries of patients with ST-segment-elevation myocardial infarction during primary percutaneous coronary intervention (29).

In a large cohort of 707 consecutive patients with symptomatic CAD, the association of platelet-derived PCSK9 and platelet aggregation was evaluated demonstrating that PCSK9 inhibition significantly reduced platelet-dependent thrombus formation (46). They observed a significant reduction of the thrombus area in whole blood samples treated with anti PCSK9 antibodies. In addition, they observed that circulating platelets expressed and released PCSK9 in high amount promoting platelet aggregation and thrombus formation, monocyte migration and differentiation into macrophages/foam cells, thus further contributing to the atherosclerotic process. In patients with atrial fibrillation, PCSK9 induced platelet activation by Nox2 via binding with CD36 receptor (47).

However, the clinical relevance of measuring PCSK9 circulating levels as a predictor or qualifier for cardiovascular (CV) risk estimation remains unanswered. Several studies did not associate PCSK9 levels with the number of events (48), as was in the case of acute coronary syndrome patients in which PCSK9 was associated with inflammation and hypercholesterolaemia without predicting the mortality at one year (49); conversely, different conclusions were reached by others (50, 51).

## 2. Proteomic and lipidomic strategies

The advances in mass spectrometry (MS) technologies could allow gaining additional information on various PCSK9 targets involved in the development and progression of cardiac and cardiovascular complications. Mass spectrometry coupled with liquid chromatography (LC-MS) is a powerful and sensitive approach that can be used to identify and quantify proteins in complex biological samples, providing a global proteome view of the sample (untargeted proteomics) or a targeted detection of a specific protein of interest.

Proteomics methods have evolved from gel-based techniques to gel-free MS-based approaches, obtaining convenient and in-depth proteome coverage (52). PCSK9 is a low abundant plasma protein and, for this reason, a pre-treatment step such as immunoenrichment or solid-phase extraction (SPE) before the MS analysis is often required to allow an accurate detection. Quantitative MS analysis can be performed both using a label-free approach, mainly applied for a discovery analysis (53), and also using protein/peptide labelling with stable isotopes (54). Label-based quantification involves the artificial labelling of peptides or proteins before liquid chromatography-tandem mass spectrometry (LC-MS/MS), which generates a mass difference between the endogenous peptide and its labelled counterpart, and their intensity ratio allows a measurement of the protein abundance (55).

Targeted proteomics, instead, limits the analysis to a specific number of proteins for screening or validation of candidate protein biomarkers that could be useful for diagnosis, prognosis, and characterization of pathophysiological mechanisms. Multiple reaction monitoring (MRM) is an example of targeted MS-based strategy, which guarantees high sensitivity and selectivity, giving reproducible results at very low levels of quantitation (56). Furthermore, due to its great high-throughput features, it is possible to use this targeted MS technique in several proteomics areas to understand better the abundances and activities of individual proteins, as well as to validate their potential role as biomarkers.

In this context, Croyal et al. measured plasma PCSK9, as an example of a low abundant protein, by LC-MS/MS and compared the results to those obtained by using standard enzyme-linked immunosorbent assay (ELISA) (57). The authors showed that the calculated PCSK9 plasma concentrations were not statistically different between the two methodologies. They also found that a SPE clean up allowed to avoid a sample concentration by immunoaffinity before sample proteolysis, and the subsequent analysis of a PCSK9 proteotypic peptide, by using MRM, successfully provided a targeted quantification of plasma PCSK9. A proteomics approach enables to discriminate among different forms of PCSK9. The PCSK9 PAC-qMS assay allows to measure seven PCSK9 peptides based on prodomain, the catalytic domain before and after furin cleavage, the hinge-, the cysteine- and histidine-rich domain, and the C-terminal domain encompassing S688 in both its unmodified and phosphorylated form. By using this method, it has been possible to demonstrate that analysis of prodomain and the phosphorylated state of S688 may represent novel cardiovascular risk biomarkers (58).

MS is successfully applied also in lipidomics for the study of cellular lipids on a large scale. Lipids are highly complex molecular species with important roles in cellular functions and several metabolic pathways (59). Therefore, the identification and accurate quantification of lipids provide a powerful tool to understand the mechanism of lipid-related diseases and disorders (59). A typical MS-based workflow for lipidomics of biological samples includes sample preparation, MS-based acquisition and data processing. The major challenge in lipidomics is lipid extraction from samples and several strategies were developed in the past decades due to the different nature of lipid species (59, 60). However, there is no extraction method that can completely extract all lipid species, and several preparative modifications have been made to improve and increase the efficiency of extracting specific types of lipids (61). Certainly, the aim is to optimize a simple method for extracting all major classes of lipids by using a minimal amount of sample and for obtaining a high recovery rate and reproducibility. The typically used methods for lipidomic analysis are nuclear magnetic resonance spectroscopy (NMR), gas chromatography (GC) after derivatization of polar groups, and LC-MS. The advances in MS with the development of highly sensitive MS detectors allowed higher resolution, sensitivity, and throughput (59). Furthermore, along with the analysis of lipids with LC-MS, it is possible to perform either a direct analysis without previous chromatographic separation with MS imaging (MSI) or a direct infusion of the total lipid extracts using shotgun lipidomics. Likewise, to proteomics analysis, MS-based lipidomics can be performed with untargeted or targeted objectives. For untargeted lipidomics, non-selective extraction protocols are required that can extract all detectable lipid classes in a sample without contaminating non-lipid molecules such as proteins and carbohydrates (60). Instead, the main focus of targeted lipidomic analysis is the analysis of specific types of lipids and thus the selection of the extraction method is crucial to obtain the correct recovery.

In addition, solid phase extraction is a purification strategy that can help in the isolation of selected lipids or the enrichment of small lipid classes using specific cartridges packed with different sorbents able to selectively hold the desirable fractions and to let undesirable compounds pass through (59).

## 3. Agnostic evaluation of PCSK9 effects by proteomics and lipidomics

**Figure 2:**
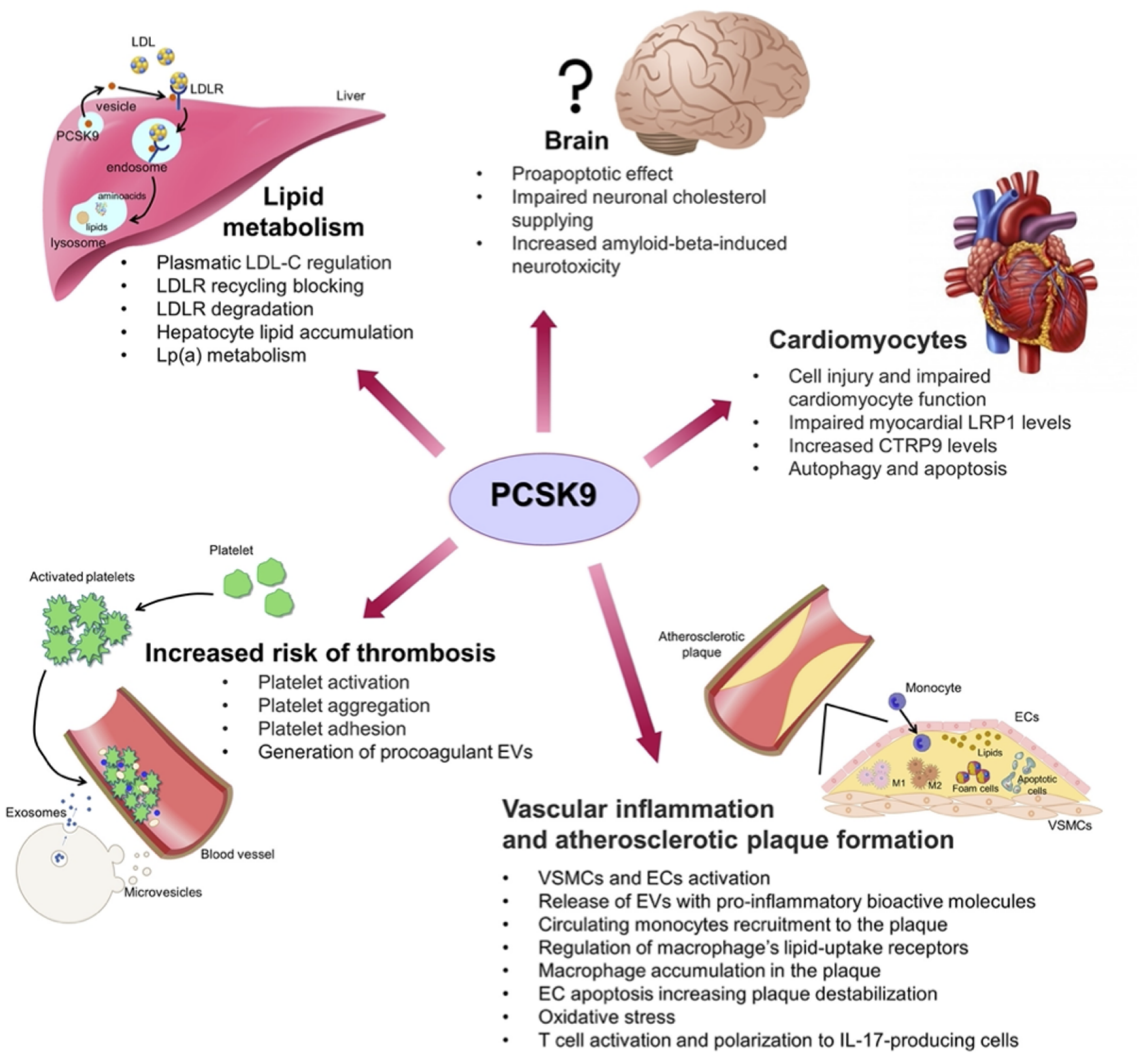
Summary of the non-classic effects played by PCSK9 on various cell types in different organs: liver and lipid metabolism; brain; cardiomyocytes; vascular system with inflammation, atherosclerotic plaque formation and increased risk of thrombosis; CTRP9, C1q/tumour necrosis factor-related protein 9; ECs, endothelial cells; EVs, extracellular vesicles; LDL, low-density lipoprotein; LDL-C, LDL cholesterol; LDLR, low-density lipoprotein receptor; LRP1, low density lipoprotein receptor-related protein 1; M1, macrophages type 1; M2, macrophages type 2; VSMCs, vascular smooth muscle cells.

Besides regulating LDL-C homeostasis, PCSK9 non-cholesterol-related processes have emerged since its discovery. Mainly secreted from the liver, PCSK9 is expressed in many tissues (e.g., intestine, pancreas and central nervous system) (Figure 2). Thus, to unveil new molecular targets of PCSK9 is mandatory to foresee possible drawbacks related to the long-term PCSK9 pharmacological inhibition.

### 3.1. PCSK9 and atheroma formation

A deep molecular phenotyping of samples from homozygous carriers can help to discriminate between silent and functional variants. Combining whole-genome sequencing with proteomics and metabolomics have allowed to discover a homozygous *PCSK9* genetic variant (rs746442570) which was associated to low PCSK9 protein levels and LDL-C levels (62).

Denis et al. performed a quantitative MS analysis with a metabolic stable isotope labelling by amino acids in cell culture (SILAC) to study proteomic changes occurring in different subcellular compartments of the human hepatocyte cell line (HuH7) after a stable overexpression of a gain-of-function PCSK9 membrane-bound chimaera (PCSK9-V5-ACE2) (63). After SILAC labelling, subcellular fractionation was performed before the protein separation by Sodium Dodecyl Sulphate—PolyAcrylamide Gel Electrophoresis (SDS-PAGE), tryptic digestion and LC-MS/MS analysis. As mentioned before, gain- and loss-of function variants in human PCSK9 modulate the levels of circulating LDL (31). More than 5,700 peptides were quantified in the lysosomal/endosomal, Golgi and endoplasmic reticulum compartments, among which 327 showed significant changes in the expression levels following stable overexpression of PCSK9-V5-ACE2 in comparison to the control cells transfected with a mock vector. These proteins were involved in multiple functions including cytoskeletal organization, vesicle transport, membrane receptor recycling, lipid and cholesterol homeostasis, cell signalling and protein folding. In particular, it was confirmed a significant downregulation of the levels of EH-domain binding protein 1 (EHBP1), a protein involved in receptor recycling from internal vesicles back to the cell surface. This suggests a reduced recycling of the LDLR and its relocation to the late endosome and lysosome for degradation (63). Moreover, several proteins of the Rab family of GTPases, that control vesicle transport, vesicle movement along cytoskeleton, and membrane networks, showed both higher and lower expression by PCSK9-V5-ACE2 overexpression in HuH7 cells (63). Among the proteins that were upregulated upon PCSK9-V5-ACE2 overexpression, A-kinase anchor protein-12 (AKAP-12) is a potent scaffold protein that interacts with many signalling factors and facilitates the internalization and the recycling of the β2-adrenergic receptor through calcium-dependent interactions (63). Its overexpression leads to an upregulation of the expression of sterol regulatory element-binding protein-2 (SREBP-2) and liver 3-hydroxy-3-methyl-glutaryl-CoA reductase (HMGCoA), resulting in an enhancement of cholesterol efflux from hepatic cells (64). Overall, this study found that an endosomal-related protein was affected by PCSK9 providing insight into novel functions and pathways affected by PCSK9 and unrelated to LDLR processing.

### 3.2. PCSK9 and cardiovascular diseases

In the context of CVDs, PCSK9 is overexpressed in the ischaemic mouse hearts, mostly in the region bordering the infarct area, and promotes the development of infarct size, cardiac contractile function, and autophagy (65). Cultured mouse cardiomyocytes were examined for the correlation between myocardial ischaemia and PCSK9 expression and autophagy, and PCSK9 levels increased when cardiomyocytes were in hypoxia condition, in a hypoxia duration-dependent manner. Moreover, proteins from hearts and primary cardiomyocytes were purified and analysed by WB demonstrating that deletion of hypoxia inducible factor-1 α (HIF-1α) significantly reduced the expression of PCSK9, microtubule-associated protein 1 light chain 3 (LC3), and beclin-1 during ischaemia. Therefore, a possible association between PCSK9 and cardiac diseases and their risk factors is now well-defined in the literature. Independently of LDL-C regulation, PCSK9 exerts various effects in the heart and its higher levels promote reactive oxygen species production, excessive autophagy and apoptosis in cardiomyocytes leading to a cardiac insufficiency following the acute myocardial infarction (66). In the attempt to reduce the CVD burden driven by LDL-C, the use of inhibitors of PCSK9 has provided a very powerful therapy for patients at highest risk (67). Despite the well-demonstrated benefits of statins, a large number of patients does not reach adequate levels of LDL-C recommended by guidelines, as well as blood triglyceride-rich lipoproteins, or lipoprotein(a), thus still remaining at significant risk of CVD. For this reason, alternative cholesterol lowering approaches to use in combination with statin, such as inhibitors of PCSK9, have been developed to obtain better efficacy and tolerability.

Among the risk factors accounting for CVD is now recognized that lipid and lipoprotein abnormalities play a key role in this process. So said, the implication of PCSK9 inhibition on lipid classes, besides LDL, has been only recently investigated. Hilvo et al. performed a lipidomic analysis in patients with established coronary heart disease after administration of a RG7652, a fully human PCSK9 monoclonal antibody, to characterize the lipidome alterations of plasma and major lipoprotein particles (67). Lipoprotein fractions were isolated by sequential density ultracentrifugation and analysed in MS to obtain a lipidomic profiling. A significant reduction of sphingolipids, cholesteryl esters and free cholesterol was found in plasma upon PCSK9 inhibition, as was for lipids in lipoprotein classes. A global decrease of total lipid species was reported in LDL, very low-density lipoprotein/intermediate low-density lipoprotein (VLDL-IDL) particles, whereas high-density lipoproteins (HDL)-associated phospholipids rose (e.g., linoleic acid and arachidonic acid containing phosphatidylcholines (PCs) and phosphatidylethanolamines). In particular, the most pronounced reduction in both LDL and VLDL-IDL was reported for cholesteryl esters and sphingolipids including dihydroceramides (Cer d18:0), ceramides (Cer d18:1), globotriasoylceramides (Gb3), glucosyl/galactosylceramides (Glc/GalCer), lactosylceramides (LacCer), and sphingomyelins. In addition, in LDL fraction also PCs showed a significant decrease, as well as phosphatidylethanolamines and phosphatidylinositol.

Ceramides containing very long saturated fatty acid (22:0, 24:0 and 26:0) showed a more extensive decrease in plasma levels following inhibition of PCSK9 compared to the other ceramide species. To be noted that Cer(d18:1/24:1)/Cer(d18:1/24:0) ratios are able to predict fatal cardiovascular events in patients with stable coronary artery disease and acute coronary syndrome (68). This study was the first characterization of the lipidome of plasma and isolated lipoprotein fractions following therapy with a PCSK9 inhibitor (67).

Similarly, a ceramide- and phospholipid-based risk stratification score, known as CERT2, can successfully predict residual CVD event risk in CAD patients demonstrating an excellent and potentially clinically relevant prognostic value for future personalized medicine (67). The lipidomic profile in patients with a very high risk of atherosclerotic CVD was also evaluated by ultra-performance LC-MS (UPLC-MS) (69). In patients receiving evolocumab, besides the evaluation of total cholesterol, LDL-C, and lipoprotein(a), also changes in cholesteryl esters, triacylglycerols, and sphingomyelins were investigated by MS. Compared to individuals given atorvastatin alone, the addition of evolocumab led to a drop in sphingolipids, phospholipids, triacylglyceride, and diacylglycerol. However, it should be pointed out that this study is a two-centre study with a small cohort of selected patients (*n* = 64) and only changes in the blood lipidomic profile at short-term have been studied, not focusing also on long-term follow-up data about the effects of treatment and the potential cardiovascular impact. In addition, the authors did not evaluate the effects of atorvastatin and evolocumab on the specific lipid molecules in each lipid class, which could provide a far more comprehensive overview of what was happening on the lipidomic profile. Furthermore, the PCSK9-related genes, which could affect the therapeutic efficacy of PCSK9 inhibitors, were not identified.

### 3.3. PCSK9 and familial hypercholesterolemia

Familial hypercholesterolaemia is one of the most common inherited metabolic diseases in humans and can affect individuals from all ethnic groups. It associates with mutations in several genes that result in the inability of the liver to sufficiently remove LDL particles from the circulation. Autosomal dominant mutations in the *LDLR*, *APOB* (apolipoprotein B100) and *PCSK9* genes account for most cases of familial hypercholesterolaemia (70). Plasma lipidome of familial hypercholesterolemic patients before and during therapy with evolocumab was studied by an ultra-high-performance LC-MS (UHPLC-MS) to obtain an untargeted lipidomic analysis and to evaluate the effect of PCSK9 inhibition on whole lipid metabolism (71). After lipid extraction using a chloroform/methanol solution added with butylhydroxytoluene, lipid samples were analysed by MS and, after treatment, significant low plasma levels were observed in several lipid classes belonging to sphingomyelin, ceramide, cholesteryl ester, phosphatidylcholine, triacylglycerol and phosphatidylinositol, as compared to baseline. Instead, four classes, including three ceramides and phosphatidylinositol, were increased following evolocumab. In accordance with Hilvo et al. (67), sphingomyelin, ceramide, and cholesteryl ester were the species most affected by the treatment (71). It is possible to assume that these lipidomic changes may be due to an indirect mechanism that involved the *de novo* synthesis of sphingomyelin and ceramide through the activation of sphingomyelin synthase by oxidized LDL, thus increasing cholesterol ester production. Therefore, the upregulation of LDLR and the lipidome modifications seem to determine an improvement of vascular state in patients upon the pharmacological inhibition of PCSK9. However, further targeted analysis, based on lipid fraction isolation, is needed to strengthen these conclusions and the identification of antibodies against oxidized LDL will be necessary to deepen the role of oxidized LDL in the modulation of ACS.

### 3.4. PCSK9 and new-onset diabetes

In the last few years, numerous studies have shown that *PCSK9* LOFs variants are associated with higher fasting plasma glucose levels, possibly leading to an increased risk of type 2 diabetes (72, 73), a side effect not confirmed in clinical trials (74). Recently, Saitoski et al. investigated the role of PCSK9 in insulin-producing pancreatic cells to clarify its function and to evaluate potential other protein targets beyond LDLR (75). The authors studied PCSK9 regulation and function in the human pancreatic beta cell line EndoC-βH1, showing that PCSK9 is expressed and secreted by these cells and its expression is regulated by cholesterol, lipoproteins, and sterol regulatory element-binding protein transcription factors. Western blot analyses showed that cholesterol and lipoproteins, except for oxidized LDL that had no effects, reduced intracellular and secreted PCSK9 levels. Following PCSK9 knockdown by using a siRNA approach, several deregulations in the transcriptome, proteome, and secretome of these cells were detected, along with rises in basal and glucose-stimulated insulin secretion (75). Ingenuity pathway enrichment analysis showed impaired mitochondrial function, which is essential for insulin secretion by beta cells, and differential expression of several markers associated with glucose-stimulated insulin secretion. By using Tandem Mass Tag labelling and data-dependent acquisition MS on cell lysates, together with other data-independent acquisition cell proteomic and secretome experiments, higher levels of the enzyme proprotein convertase subtilisin/kexin type 1 (PCSK1) involved in proinsulin to insulin processing were noticed at 72 h after PCSK9 knockdown. MHC-I complex and Golgi/ER proteins were also overexpressed. The authors also performed loss- and gain-of-function experiments in the EndoC-βH1 cells using siPCSK9 or recombinant PCSK9 treatments to discover proteins regulated by PCSK9 in human beta cells. By Western blot, they observed low levels of LDLR and very low-density lipoprotein receptor (VLDLR) only through an extracellular signalling mechanism involving exogenous PCSK9, while CD36 via an intracellular signalling mechanism. The specific mechanism of CD36 regulation by PCSK9 was not clarified by the authors but maybe involved both proteasomal and lysosomal degradation, so future experiments by using proteasome or lysosome inhibitors will be necessary to better understand the role of PCSK9 on CD36 regulation in the pancreatic β cells.

Thus, both intracellular and extracellular PCSK9 regulate β-cell fatty acid homeostasis. Finally, intracellular PCSK9 was found to modulate the cell surface expression of programmed death-ligand 1 (PDL1) and human leukocyte antigen (HLA)-ABC, which are proteins involved in cell-lymphocytes interaction. Therefore, this study confirms that PCSK9 is a regulator of multiple cell surface receptors in pancreatic beta cells (75). The authors did not discuss the potential effect of PCSK9 monoclonal antibodies or siRNA-based therapies against PCSK9 on the expression of LDLR, VLDLR, CD36, PDL1, and HLA-ABC in pancreatic β cells, as was in the case of cotreatment with statins that could modify the concentrations of these proteins.

However, a recent study by Peyot et al. reported contradictory results regarding the glucose metabolism regulation following the inactivation of PCSK9, demonstrating that in beta cells in mice the reduction of PCSK9 mRNA and protein expression did not affect glucose homeostasis and β-cell function (76). The authors created the first β-cell-specific KO of PCSK9 (βKO) and showed that both whole body *Pcsk9^−/−^* KO and β*KO* mice presented an increased cholesterol uptake, but normal glucose tolerance, insulin release in response to glucose load and insulin sensitivity as compared to their respective control wild type mice. Moreover, *ex vivo* glucose-stimulated insulin secretion was similar in wild type and KO islets both in presence or absence of fatty acids. According to these results, another study showed that liver-specific KO of PCSK9 in mice did not alter the expression of LDLR in β-cells and maintained insulin secretion and glucose homeostasis (72). Conversely, mice specifically lacking *Pcsk9* in pancreas and those with whole loss of all local PCSK9 in islets showed a moderate impairment of insulin response and glucose tolerance (73). This suggests the importance of local PCSK9 expression in pancreatic islets to maintain a β-cell function. Similarly, impairment of glucose metabolism is also variable in human subjects carrying *PCSK9* LOF variants, an effect depending on the genetic background of the studied population, in which the risk to develop diabetes is also associated to the individual degree of functional loss (77).

Therefore, several *PCSK9* LOF variants have been reported in literature with different effects on glucose homeostasis and many controversies exist (78). Some variants result in a significant increase in glucose levels and higher risk of type 2 diabetes, other variants do not cause alteration of glucose metabolism (74).

### 3.5 PCSK9 and central nervous system

Hypercholesterolemia can cause neuroinflammation in midlife and is associated with a higher risk of age-related neurological disorders including Alzheimer’s disease (79). Cholesterol is important for maintaining normal brain functions and most of it is synthesized *de novo* and separated from peripheral cholesterol through the blood-brain-barrier (80).

The role of PCSK9 in neurodegenerative disorders is still unclear and conflicting data on this subject have been recently published, as elsewhere reviewed (81) An *in vitro* study on PCSK9 modulation of human astrocytes and neurons showed an altered cholesterol metabolism and neuronal cholesterol supplying, which resulted to be significantly reduced following the PCSK9 overexpression, thus impairing the normal neuronal functions (82). Moreover, PCSK9-overexpressing cells displayed increased amyloid-beta-induced neurotoxicity compared to control cells. Although Mendelian randomization analyses and meta-analyses on early studies have hypothesized a PCSK9-related neurocognitive impairment (83–85), analyses of cardiovascular outcomes trials (CVOT) did not show a risk of neurocognitive adverse effects following the use of PCSK9 inhibitors (86, 87), actually, prolonged exposure was associated with a decreased risk (88).

The evidence that also serum cholesterol levels represent a risk factor for dementia and cognitive impairment is still unclear. Many side-chain hydroxylated metabolites of cholesterol, such as 24S-hydroxycholesterol (24S-OHC) and 27-hydroxycholesterol (27-OHC), can pass the brain-blood-barrier and are implicated in neuroinflammation (89). In particular, it was assumed that 27-OHC in the brain reduces central nervous system cholesterol synthesis and 24S-OHC production (90).

The effects of evolocumab and alirocumab on lipidome profile were evaluated in hypercholesterolemic patients responding insufficiently to maximally tolerated statin and/or ezetimibe therapy. The brain-specific oxysterols 24S-OHC and 27-OHC were assessed by isotope dilution GC-MS selected ion-monitoring (GC–MS-SIM) by using the corresponding deuterium-labelled oxysterols as internal standards, before, after 1-month and after 3-month of treatment (91). 24S-OHC ratios to cholesterol and to 27-OHC in serum or plasma are thought to be potential biomarkers for brain cholesterol metabolism (92). After 1-month of treatment, evolocumab and alirocumab significantly reduced serum total cholesterol, LDL-C and 27-OHC concentrations, whereas serum ratios of 24S-OHC to cholesterol and 24S-OHC to 27-OHC increased, reaching even higher levels within 3 months of treatment (91). The authors suggested that this specific brain cholesterol turnover was probably due to lower levels of 27-OHC in the brain, resulting in increased synthesis of brain cholesterol, higher production of 24S-OHC and its secretion across the blood-brain barrier. Therefore, this study demonstrated how brain excretion of 24S-OHC is improved upon anti-PCSK9 antibody treatment. Anyway, in this study, the cohort of hypercholesterolemic patients was small with large variability in sex, pre-treatment and PCSK9 inhibiting agent, and the percentages of increment of 24S-OHC and 24S-OHC to 27-OHC after 1-month treatment were statistically significant even if small. Of course, wider and longer-term demographic studies are needed. The measurement of 7α-hydroxy-3-oxo-4-cholestenoic acid, which is formed from the conversion of 27-OHC in the brain, could also be useful to obtain more information on brain cholesterol metabolism because this conversion may affect the inhibition of brain cholesterol synthesis by 27-OHC. Moreover, the authors would have to measure the arteriovenous difference of oxysterols 24S-OHC and 27-OHC between the internal jugular vein and an artery also in patients during PCSK9 treatment and not only in healthy normocholesterolaemic subjects to have a more complete view of the flux of these oxysterols.

## 4. Proteomics to identify PCSK9 targets

The major critical function of PCSK9 deduced from human and mouse studies, as well as cellular and structural analyses, is its role in increasing the levels of circulating LDL-C, via its ability to enhance the sorting and escort of the LDLR to lysosomes (93). However, in this complex scenario, many unsolved questions remain, e.g., it was demonstrated that PCSK9 lacking its C-terminal Cys/His-rich domain was still able to regulate LDLR internalization, but PCSK9-LDLR complex did not follow the endosome/lysosome pathway for degradation suggesting the presence of additional interactors (94, 95). Therefore, it is important to investigate the presence of additional membrane-bound or secreted cellular proteins involved in the LDLR internalization and degradation mediated by PCSK9.

By affinity purification and shotgun LC-MS/MS analysis, Xu et al. identified 22 potential PCSK9 binding proteins (96), including endoplasmic reticulum-localised proteins, proteins associated with the ubiquitination pathway (e.g., cellular inhibitor of apoptosis protein 1 and TNF receptor-associated factor 2), mitochondrial carriers, and molecular chaperones. In particular, they focused the attention on cellular inhibitor of apoptosis protein 1 (c-IAP1) showing that this protein binds and processes PCSK9 (96). Indeed, in carriers of the S127R, a gain-of-function variation, PCSK9 was not able to bind to c-IAP1, impairing its autocatalytic activity. However, the specific mechanism by which the S127R mutant proprotein stimulates LDLR degradation causing hypercholesterolaemia still remains unresolved. Therefore, further clarification of this aspect will be necessary.

Using a siRNA-c-IAP1 construct to knock down endogenous *c-IAP1* in a human T-Rex-293 stable cell line overexpressing FLAG-tagged wild-type PCSK9, the same authors showed a significant increase of pro-PCSK9 in comparison to the functionally mature PCSK9 that is the predominant form in non-silencing RNA control; this suggests that c-IAP1 impairs PCSK9 processing and autocatalytic cleavage. In the same study, there were found lower levels of secreted mature PCSK9 protein in the medium from c-IAP1 siRNA treated vs. control samples and a higher concentration of pro-PCSK9 protein aggregates in c-IAP1 siRNA cells. In *c-IAP1 null* mouse embryonic fibroblasts, there was a reduction in the secreted mature form of PCSK9 together with a significant increase in the levels of LDLR. Finally, the study reported a role of c-IAP1 in the PCSK9 ubiquitination mediated by the only lysine-27, which led to the lysosomal localization of the PCSK9/LDLR complex and its possible degradation (96). Therefore, this study provided new insights into the roles of c-IAP1 in regulating the activities of PCSK9, giving a clue to develop more efficient treatments against cardiovascular and infectious diseases.

Ly et al. performed a co-immunoprecipitation of PCSK9 combined with an UHPLC-MS/MS analyses in HepG2 secretome to identify PCSK9-binding proteins able to control the PCSK9-LDLR complex formation and LDLR degradation (97). The authors identified a total of 42 secreted proteins and, among the proteins linked to cholesterol metabolism, many potential PCSK9 interactors were selected, such as glypican-3 (GPC3), matrilin-3, phospholipid transfer protein, fibrinogen-like 1, tissue factor pathway inhibitor, and plasminogen activator inhibitor-1. By silencing each of these proteins in HepG2 cells, they demonstrated also that only GPC3 and phospholipid transfer protein were able to significantly increase LDL uptake and led to higher total LDLR protein levels in these cells compared to control cells (97). Focusing on GPC3, data from this study showed that PCSK9 Cys/His-rich domain is not involved in the interaction with extracellular GPC3, which instead happens with the PCSK9 prodomain and/or catalytic subunit. Moreover, to investigate whether intracellular GPC3 is also implicated in the interaction with the PCSK9-LDLR complex, the authors analysed both HepG2 and Huh7 cells and concluded that GPC3 interacts with pro-PCSK9 and immature LDLR intracellularly most likely in the endoplasmic reticulum. In conclusion, the findings led the authors to demonstrate a competitive interaction between GPC3 and PCSK9 which can reduce the PCSK9 extracellular activity on LDLR degradation (97). However, further studies are needed to verify which is the exact region or residues involved in the GPC3-PCSK9 interaction, because the authors in this study did not deepen this structural aspect.

Another aspect to be worth considering is that the understanding of the protein-protein interaction that governs the PCSK9-LDLR interaction could be fundamental to developing new therapeutic strategies in the near future.

A combination of affinity chromatography, high-resolution LC-MS/MS proteomics, gene expression, and ELISA assays (98) allowed to identify three proteins, alpha-1-antitrypsin, alpha-1-microglobulin/bikunin precursor, and apolipoprotein H that interact with PCSK9. Alpha-1-antitrypsin and apolipoprotein H showed a higher expression both at the mRNA and protein levels in human hepatic C3A cells treated with MITO+ medium, which is a medium able to significantly affect PCSK9 function lowering PCSK9/LDL receptor complexes levels as compared to cells exposed to standard medium. Conversely, alpha-1-microglobulin/bikunin precursor did not statistically change its expression in response to the treatment. In particular, the authors noticed that under MITO+ condition the secretion of apolipoprotein H was lower, while its internalization was increased. In addition, they suggested an interaction with PCSK9 at the plasma membrane and within the endosome for alpha-1-antitrypsin and alpha-1-microglobulin/bikunin precursor, while apolipoprotein H may interact with PCSK9 mainly in the endosome (98). Previous evidence described the formation of complexes between oxidized alpha-1-antitrypsin and LDL in atherosclerotic lesions, although their mechanisms underlying the formation and clinical significance are still unclear (99). Similarly, apolipoprotein H is an important player in atherosclerotic plaque and contributes to atherosclerosis progression by binding oxidized LDL (100, 101).

In contrast, alpha-1-microglobulin/bikunin precursor has not yet been associated with atherosclerosis, but no evidence suggests otherwise since α1-microglobulin has been suggested to be involved in the protection against atherosclerosis, inhibiting the oxidation of LDL and counteracting heme toxicity and reactive oxygen species (102).

In humans, PCSK9 circulates in a free and lipoprotein-bound form (103), possibly bound to LDL (104), lipoprotein(a) [Lp(a)] (105) and HDL (106). Burnap et al. evaluated the associations between PCSK9 and apolipoproteins in fasted plasma from two prospective, community-based cohort studies by nuclear magnetic resonance spectroscopy and targeted MS analysis (107). A significant positive correlation was observed between plasma PCSK9 levels and the particle number of small dense HDL, as well as a strong association between PCSK9 and apolipoprotein C3 (apoC3), an inhibitor of lipoprotein lipase that can hydrolyse plasma triglycerides. In addition, the authors investigated the PCSK9 kinetics in healthy volunteers during postprandial lipemia and characterized the HDL composition by label-free MS. They confirmed a decrement of PCSK9 levels during the postprandial lipemia peak within 5 h compared to the fasted condition, while a negative correlation was found between the postprandial PCSK9 and triglyceride responses. The postprandial reduction of HDL-bound PCSK9 was also confirmed by proteomics, and a decrease of apoC3 from HDL was observed at the same time, as well as an increase of ApoA1 and inflammatory-related proteins, respectively, at 8 and 4 h postprandially. Using crosslinking MS to study plasma and HDL interactome, PCSK9 was identified to interact with HDL through apoA1 binding, and the further quantitative analysis of HDL proteome and lipidome in a cohort of patients with CAD vs. control subjects revealed a strong distinct cluster of PCSK9, phospholipid transfer protein, clusterin, and apolipoprotein E that was statistically altered by sex and positively associated with sphingomyelin content (107). Finally, the authors determined whether HDL was able to alter PCSK9 function, demonstrating that HDL promoted PCSK9-driven LDLR degradation through the modulation of PCSK9 cellular uptake and multimerization. Therefore, this study demonstrated that the interaction between PCSK9 and HDL impairs PCSK9 functionality. In future studies, it will be interesting to unravel the mechanisms between PCSK9 function and lipoprotein binding to evaluate the possibility to use small molecule inhibitors instead of monoclonal antibodies to inhibit PCSK9 activity with higher therapeutic efficacy and lower costs.

## 5. Proteomics to identify PCSK9-driven changes in extracellular vesicles

In search of new mechanisms linking PCSK9 with cardiovascular risk, the role of extracellular vesicles (EVs) should be considered. EVs are membrane-bound particles released by cells in biological fluids, both in physiological and pathological contexts and seem to participate in each stage of the atherosclerotic process. They carry a cargo composed of microRNAs, proteins, lipids, and organelles that vary according to environmental stimuli and risk factors (108). The content of ceramides and phosphatidylcholines in plasma EVs assessed in preoperative state was independently associated with an increased 3-year risk of major adverse cardiac events in patients undergoing carotid endarterectomy (109). PCSK9 influences the release of EVs, in particular those derived from macrophages (CD14^+^), endothelium (CD105^+^), and neutrophils (CD66^+^) (110). PCSK9 seems to mediate the generation of procoagulant EVs, a process that could contribute to an increased prothrombotic status in patients with CVDs (111).

So said, it becomes of interest to explore the relationship between PCSK9 and the release of EVs from cells relevant to the atherosclerotic process to hypothesize the potential use of these EVs as a pharmacological vehicle. As an example, in patients with obesity, PCSK9 has a key role on the release of EVs-derived from different atherosclerotic components (i.e., platelets, monocytes/macrophages, endothelium, and neutrophils) and EVs-derived miRNA linked to inflammation and atherosclerosis (112). In particular, the PCSK9 effect on platelet-derived EVs was affected by platelet count in a significant and negative way.

EVs are vehicles of bioactive molecules involved in several atherosclerotic processes, among which lesion development, plaque progression, hemostasis and thrombin regulation (113). In this regard, a recent study (111) confirmed the PCSK9-mediated generation of procoagulant EVs from human mononuclear cells (PBMCs) and THP-1 cells via TLR4/NF-κB activation, thus increasing the prothrombotic status in patients with CVDs. The pharmacological inhibition of PCSK9 with evolocumab showed a reduction of PCSK9-induced EV generation and extracellular-bound tissue factor. Therefore, PCSK9 stimulates the generation of prothrombotic EVs by cells of monocytic lineage, and these EVs are characterized by a TF-dependent procoagulant activity. These data demonstrate the important role of PCK9 in the atherothrombotic process.

In addition, PCSK9 seems to confer procalcific and inflammatory properties to EVs released by VSMCs, which are cells relevant for the atheroma formation (114, 115). Since PCSK9 is expressed and secreted as functionally active by VSMCs, an association between PCSK9 and the release of EVs was recently described (110). PCSK9 is released in significant amounts from of VSMCs, more than from endothelial cells (116), and it is abundantly expressed in VSMCs of human atherosclerotic lesions and abdominal aortic aneurysm tissues (117) suggesting its potential role in plaque instability and increased cardiovascular events. The impact of PCSK9 on the composition of EVs released from human VSMCs and how these EVs regulated the intercellular communication among atherosclerotic components have been the leverage to study a model of human VSMCs overexpressing PCSK9 (VSMC^PCSK9^) (114). Using untargeted proteomic analysis, VSMC^PCSK9^-EVs showed a different and specific cargo of proteins compared to EVs derived from their counterpart released by VSMCs wildtype, mainly represented by extracellular matrix structural constituents that are involved in different aspects of cardiovascular pathophysiology. The isolated EVs were then subjected to functional experiments by using both *in vitro* and *in vivo* models, demonstrating that when VSMCs expressed higher levels of PCSK9, the released EVs carried pro-inflammatory bioactive molecules. The impact of EVs on pro-inflammatory markers in endothelial cells, monocytes and monocyte-derived macrophages was evaluated and a significantly raised expression of adhesion molecules was observed in endothelial cells exposed to VSMC^PCSK9^-EVs, as well as an increase of pro-inflammatory cytokines and proteins in monocytes and macrophages. All these proteins are clear promoters of vascular inflammation and atherosclerosis initiation or progression. Using a label-free mass spectrometry-based approach, also the secretome of monocytes exposed to EVs was analysed to confirm these data, reporting proteins involved in immune response and immune effector processes. Moreover, VSMC^PCSK9^-EVs enhanced the migratory capacity of monocytes and glycolytic activity while decreasing oxidative phosphorylation. Macrophages showed a reduced migratory capacity, as a mirror of their accumulation in atherosclerotic plaques, and higher oxLDL-derived cholesterol uptake. To better understand the role of macrophages as mediators of the pro-inflammatory state, when VSMC^PCSK9^-EVs were microinjected into an *in vivo* model of zebrafish embryos, there was an increased recruitment of macrophages toward the local injection site (114). This study highlighted the pro-inflammatory role of PCSK9 through the content of EVs released from VSMCs, pointing out the cholesterol-independent function played by PCSK9 in atheroma formation. In addition, the mitochondrial dysfunction following the treatment of monocytes with VSMC^PCSK9^-EVs confirmed the key role of mitochondria in the pathogenesis of inflammatory diseases such as atherosclerosis, thus representing a potential target for therapeutic agents.

## 6. Future perspectives

Although the clinical benefits of PCSK9 inhibition are clear, large gaps remain in the understanding of PCSK9 biology (e.g., molecular mechanisms that regulate circulating PCSK9 levels). Within this context, advancement in high-throughput approaches (e.g., proteomics and lipidomics) can allow to unravel unsuspected pathways providing knowledge on possible new therapeutic targets (Table 1). In the era of personalized medicine, identification of specific biomarkers (e.g., the phosphorylated form of PCSK9) may reveal important insights in the pathophysiology of CVDs. Considering that improvements in risk stratification is fundamental to reduce the burden of CVDs, the assessment of the proteins’ expression in a specific state and the evaluation of how these proteins change over time can identify patients at greatest risk of CV events. Although these approaches hold the promise to be assessed in a high-throughput routine, several limitations (e.g., cost-effectiveness, rigorous prospective studies, validation in rigorous clinical studies) need to be addressed before a wider clinical use (118).

**Table 1:** Summary of the main proteomic and lipidomic studies on the molecular changes associated with pleiotropic effects of PCSK9.

Sample type	Matrix	Experimental design	Analytical techniques	Pathways involved	Main proteomic and/or lipidomic targets	Reference
Human hepatocyte cell line HuH7	Cells	PCSK9 gain-of-function	SILAC labelling, SDS-PAGE, LC-MS/MS	Cytoskeletal organization, vesicle transport, membrane receptor recycling, lipid and cholesterol homeostasis, cell signalling and protein folding	EH-domain binding protein 1, Rab family of GTPases, and A-kinase anchor protein-12	Denis et al. (63)
Human pancreatic beta cell line EndoC-βH1, HepG2 and HEK293T, primary hepatocytes, human islets	Cells	PCSK9 knockdown, and loss- and gain-of-function experiments	Tandem Mass Tag labelling, LC-MS/MS, WB	Basal and glucose-stimulated insulin secretion, β-cell fatty acid homeostasis, cell-lymphocytes interaction	PCSK1, MHC-I complex and Golgi/ER proteins, LDLR, VLDLR, CD36, PDL1, and HLA-ABC	Saitoski et al. (75)
Mouse hearts and primary cardiomyocytes	Cells/ tissues	PCSK9 loss-of-function, and PCSK9 overexpression	WB	–	Hypoxia inducible factor-1 α, microtubule-associated protein 1 light chain 3, beclin-1, AMP-activated protein kinase, and ataxia-telangiectasia mutated serine/threonine kinase	Ding et al. (65)
Non-diabetic patients with established coronary heart disease	Plasma	PCSK9 inhibitor (monoclonal antibody RG7652)	LC-MS /MS	–	Sphingolipids, cholesteryl esters, free cholesterol, phospholipids, ceramides and lipid composition of the lipoproteins	Hilvo et al. (67)
Patients with a very high risk of ASCVD	Serum	PCSK9 inhibitor (Evolocumab)	UPLC-MS	–	Phospholipids, cholesteryl esters, free cholesterol, triacylglyceride, diacylglycerol, sphingolipids, LDL-C, and Lp(a)	Huang et al. (69)
Patients with FH	Plasma	PCSK9 inhibitor (Evolocumab)	UHPLC-MS	–	Sphingomyelin, ceramides, cholesteryl ester, phosphatidylcholine, triacylglycerol, and phosphatidylinositol	Anesi et al. (71)
Patients with hypercholesterolemia	Serum	PCSK9 inhibitors (Evolocumab and Alirocumab)	Isotope dilution GC-MS selected ion-monitoring	–	Total cholesterol, LDL-C, 24S-hydroxycholesterol and 27-hydroxycholesterol and their ratios to cholesterol	Lutjohann et al. (91)
Human T-Rex-293 stable cell line, and mouse embryonic fibroblasts	Cells	PCSK9 gain-of-function	Immunoprecipitation, WB, SDS-PAGE, LC-MS/MS, LC/MALDI/MS/MS	Ubiquitination pathway	Cellular inhibitor of apoptosis protein 1, endoplasmic reticulum-localised proteins, mitochondrial carriers, and molecular chaperones	Xu et al. (96)
HepG2 and Huh7 cells	Cells	PCSK9 overexpression	Immunoprecipitation, WB, and UHPLC-MS/MS	Cholesterol metabolism	Glypican-3	Ly et al. (97)
Human hepatic C3A cells	Cells	PCSK9 overexpression	Affinity chromatography, WB, LC-MS/MS	–	Alpha-1-antitrypsin, alpha-1-microglobulin/bikunin precursor, and apolipoprotein H	Melendez etal. (98)
Healthy volunteers, patients with incident primary CVD events (defined as myocardial infarction, ischaemic stroke, or vascular death), patients with coronary artery disease, and HepG2 cells	Plasma/cells	PCSK9 overexpression	Nuclear magnetic resonance spectroscopy, size-exclusion chromatography, immuno-isolation, crosslinking MS, label-free and Tandem Mass Tag labelling, LC-MS/MS	–	Apolipoprotein C3, and apolipoprotein A1	Burnap et al. (107)
Human VSMCs, endothelial cell line EAhy926, THP-1 monocytes and THP-1-derived macrophages, J774 macrophages, and zebrafish embryos	Cells/EV	PCSK9 overexpression	WB, LC-MS^E^	Extracellular matrix composition, lipoprotein particle receptor binding, very-low-density lipoprotein particle receptor binding, signalling receptor binding, adhesion, inflammation, immune response, immune effector processes	-–	Greco et al. (114)

ASCVD, atherosclerotic cardiovascular disease; CVD, cardiovascular disease; EVs, extracellular vesicles; FH, Familial Hypercholesterolemia; GC-MS, gas chromatography-mass spectrometry; GOF, gain-of-function; HLA-ABC, human leukocyte antigen-ABC; LC-MS/MS, liquid chromatography-tandem mass spectrometry; LC-MS^E^, liquid chromatography-mass spectrometry in data-independent analysis mode; LDL-C, LDL cholesterol; LDLR, low-density lipoprotein receptor; Lp(a), lipoprotein(a); MALDI, Matrix-Assisted Laser Desorption/Ionization; PCSK1, proprotein convertase subtilisin/kexin type 1; PDL1, programmed death-ligand 1; SDS-PAGE, Sodium Dodecyl Sulphate-PolyAcrylamide Gel Electrophoresis; SILAC, stable isotope labelling by amino acids in cell culture; UHPLC, ultra-high-performance LC; UPLC, ultra-performance LC; VLDLR, very low-density lipoprotein receptor; VSMCs, vascular smooth muscle cells.
